# The *RHOA* Mutation G17V Does Not Lead to Increased Migration of Human Malignant T Cells but Is Associated with Matrix Remodelling

**DOI:** 10.3390/cancers15123226

**Published:** 2023-06-17

**Authors:** Katrin Merk-Ahmad, Julia Bein, Sonja Scharf, Hendrik Schäfer, Tobias Bexte, Evelyn Ullrich, Andreas G. Loth, Nadine Flinner, Tina Senff, Olga Schneider, Martin-Leo Hansmann, Matthieu Piel, Björn Häupl, Thomas Oellerich, Emmanuel Donnadieu, Sylvia Hartmann

**Affiliations:** 1 Dr. Senckenberg Institute of Pathology, Goethe University, 60590 Frankfurt am Main, Germany; katrin.merk-ahmad@gmx.de (K.M.-A.); julia.bein@kgu.de (J.B.); nadine.flinner@kgu.de (N.F.); olgag.biol@gmail.com (O.S.); 2 Frankfurt Institute for Advanced Studies, 60438 Frankfurt am Main, Germany; sonja.scharf@med.uni-frankfurt.de (S.S.); henneck@gmail.com (H.S.); m.l.hansmann@em.uni-frankfurt.de (M.-L.H.); 3 Molecular Bioinformatics, Goethe University Frankfurt am Main, Robert-Mayer-Str. 11-15, 60325 Frankfurt am Main, Germany; 4 Institute of General Pharmacology and Toxicology, Goethe University Frankfurt am Main, Theodor-Stern-Kai 7, 60590 Frankfurt am Main, Germany; 5 Department for Pediatrics, University Hospital Frankfurt, Goethe University, 60590 Frankfurt am Main, Germanyevelyn.ullrich@kgu.de (E.U.); 6 Experimental Immunology and Cell Therapy, Department of Pediatrics, Goethe University, 60528 Frankfurt am Main, Germany; 7 Frankfurt Cancer Institute, Goethe University, 60590 Frankfurt am Main, Germany; thomas.oellerich@kgu.de; 8 University Cancer Center (UCT) Frankfurt, University Hospital, Goethe University, 60590 Frankfurt am Main, Germany; 9 German Cancer Consortium (DKTK), Partner Site Frankfurt/Mainz, 60528 Frankfurt am Main, Germany; 10 Department of Otolaryngology, Head and Neck Surgery, University Hospital Frankfurt, 60590 Frankfurt am Main, Germany; andreasgerman.loth@kgu.de; 11 Institute of Pathology and Molecular Pathology, Helios Klinikum Wuppertal, 42283 Wuppertal, Germany; tina.senff@helios-gesundheit.de; 12 Institut Curie and Institut Pierre Gilles de Gennes, CNRS, UMR 144, PSL Research University, 75005 Paris, France; matthieu.piel@curie.fr; 13 Department of Internal Medicine 2, Goethe University Hospital, 60590 Frankfurt am Main, Germany; b.haeupl@dkfz-heidelberg.de; 14 German Cancer Consortium, German Cancer Research Center, 69120 Heidelberg, Germany; 15 Institut Cochin, INSERM U1016, CNRS UMR 8104, Université Paris Cité, 75006 Paris, France; emmanuel.donnadieu@inserm.fr

**Keywords:** angioimmunoblastic T-cell lymphoma, RHOA mutation G17V, movement, live cell imaging

## Abstract

**Simple Summary:**

The aim of this study was to understand if the presence of a RHOA-G17V mutation in angioimmunoblastic T-cell lymphoma is responsible for the typical clinical presentation with early dissemination of tumor cells. Overexpression of RHOA-G17V in malignant T cells did not enhance their migration capacity neither in microfluidics microchannels, 3D collagen gel or primary human lymphoid tissue. In contrast, RHOA-G17V overexpressing cells from the HH cell line presented increased numbers of cells surrounded by cleaved collagen. Primary AITL cases showed a correlation between RHOA-G17V mutation allele frequency and collagen fibrosis of the tissue, suggesting that mechanisms leading to extracellular matrix degradation contribute to the early spread of AITL tumor cells.

**Abstract:**

Nodal T-follicular helper cell lymphoma, angioimmunoblastic-type (AITL), is characterized by constitutional symptoms, advanced-stage disease, and generalized lymphadenopathy. A genetic hallmark of this lymphoma is the frequent occurrence of the *RHOA* mutation G17V in neoplastic cells, which is observed in around 60% of patients. Because *RHOA* is involved in both T-cell receptor downstream signalling and cell migration, we hypothesized that the characteristic presentation of AITL could be the result of enhanced tumor cell migration. Therefore, this study aimed to elucidate the impact of the RHOA variant G17V on the migration of neoplastic T cells. We transfected the T-cell lymphoma cell lines HH and HuT78 to stably express the RHOA-G17V variant. RHOA-G17V-expressing T cells did not exhibit enhanced motility compared to empty-vector-transfected cells in microchannels, a 3D collagen gel, or primary human lymphatic tissue. Cells of the HH cell line expressing RHOA-G17V had an increased number of cells with cleaved collagen compared with the empty-vector-transfected cells. Therefore, we hypothesized that the early spread of AITL tumor cells may be related to remodelling of the extracellular matrix. Accordingly, we observed a significant negative correlation between the relative area of collagen in histological sections from 18 primary AITL and the allele frequency of the *RHOA*-G17V mutation. In conclusion, our results suggest that the characteristic presentation of AITL with early, widespread dissemination of lymphoma cells is not the result of an enhanced migration capacity due to the *RHOA*-G17V mutation; instead, this feature may rather be related to extracellular matrix remodelling.

## 1. Introduction

Nodal follicular T helper (T_FH_) cell lymphoma, angioimmunoblastic type (AITL), is the most common mature T-cell lymphoma in Europe [1] and North America and is categorized together with the nodal T_FH_-cell lymphomas, follicular type and not otherwise specified (NOS) under an umbrella category [2,3]. AITL was first described by Frizzera et al. in 1974 as angioimmunoblastic lymphadenopathy with dysproteinemia [4]. Clinical features of AITL include generalized lymphadenopathy, B-symptoms, splenomegaly, and a skin rash, indicating disseminated disease [5,6]. AITL differs from most other lymphomas in the severe state of patients and the involvement of multiple lymph nodes at initial diagnosis, which are frequently only moderately enlarged. Therefore, the spread and migration dynamics of the neoplastic cells of AITL seem unique. AITL is associated with immune dysregulation, high resistance to conventional chemotherapy (CHOP-like regimens) and radiotherapy, and a dismal overall survival rate of around 44% at 5 years [7]. The poor clinical outcome is likely largely due to the lack of targeted therapy.

Histological analysis has revealed three distinct infiltration patterns with either hyperplastic follicles (pattern I, according to Attygalle et al.) [8], regressed (pattern II), or completely effaced follicles (pattern III, according to Attygalle et al.) [8]. Complete structural effacement of the lymph node is frequently observed. The infiltrate consists of neoplastic T_FH_ cells with clear cytoplasm, blastic cells, arborizing vessels composed of high-endothelial venules (HEVs), and scattered large B cells, which are often infected by the Epstein–Barr virus (EBV). In contrast with many other lymphomas, AITL often infiltrates the surrounding fatty tissue (Supplementary Figure S1), suggesting that AITL cells differ from normal T cells and other lymphoma types in their migration behavior. However, extension of lymphoma infiltrates is not specific to AITL and also occurs in other types of lymphomas.

Several studies have suggested that AITL is a neoplasm derived from CD4^+^ T_FH_ cells that are normally found in reactive germinal centres, evidenced by shared gene expression signatures that are significantly enriched in T_FH_-specific genes [9,10]. Furthermore, molecular studies have identified recurrent genetic abnormalities in AITL [11,12,13,14]; *TET2*, *DNMT3A*, and *IDH2* are frequently mutated. These genes function in epigenetic regulation and are early-stage mutations that occur in the haematopoietic stem cell population. Furthermore, the small GTPase *RHOA* harbors the G17V mutation in around 60% of AITLs [11,12,13,14]. In contrast with *TET2*, *DNMT3A*, and *IDH2* mutations, *RHOA*-G17V has only been observed in mature T cells; therefore, it is considered to be a late-stage mutation. *RHOA* encodes a small GTPase protein, which is a member of the RAS family and functions as a molecular regulator of diverse cellular functions, including downstream signalling of T-cell receptors [15] and cell motility. The most well characterized effector molecules of RHOA are the kinases RHO-associated coiled-coil-forming kinase (ROCK) I and II, PRK1, PKN, and Citron, which is involved in actomyosin contraction and actin polymerization, among other functions [16,17]. Furthermore, the *RHOA*-G17V mutation is associated with the differentiation of T_FH_ cells in AITL [18,19]. Usually, *RHOA* mutations occur in AITLs at the position encoding a p.Gly17Val substitution in the GTP-binding domain [11,12]. Studies have demonstrated that RHOA-G17V inhibits GTP binding of the wild-type RHOA protein [11,12]. The frequency of the *RHOA*-G17V mutation in AITL suggests a relevant role in the pathogenesis of AITL. *RHOA* mutations are also observed in other tumors, such as adult T-cell leukemia [20], cutaneous T-cell lymphomas [21], Burkitt lymphoma [22,23], and diffuse-type gastric cancer [24]. Although the *RHOA-G17V* mutation is supposed to alter the activity of the RHOA protein, many questions remain on how it mediates tumorigenesis.

Because the neoplastic T_FH_ cells in AITL spread early and relatively easily throughout the body, the aim of the present study was to investigate the impact of the *RHOA*-G17V mutation in human malignant T cells on cell migration and cell–cell contacts.

## 2. Methods

Details on the methods can be found in the Supplemental Methods Section.

### 2.1. Cell Culture, Cloning, and Transfection

The CD4^+^ cutaneous T-cell lymphoma cell lines HH (CVCL_1280, “https://www.cellosaurus.org (accessed on 12 June 2023)”), obtained from the German Collection of Microorganisms and Cell Cultures (Braunschweig, Germany), and HuT78 (CVCL_0337, “https://www.cellosaurus.org (accessed on 12 June 2023)”), kindly provided by PD Dr. Marco Herling, University of Leipzig, were authenticated by short tandem repeat (STR) profiling within the last three years. Both cell lines were regularly tested for mycoplasma contamination.

For the generation of T-cell lymphoma cell lines that stably expressed *RHOA*-wild-type (WT) and *RHOA*-G17V, the respective sequences were cloned into a sleeping beauty vector pSBbi-GP using the sleeping beauty system, as described by Kowarz et al. [25].

First, 2 × 10^6^ cells per sample were co-transfected with 5 µg/100 µL SB-transposon-carrying DNA plasmids (RHOA-WT-pSB-bi-GP-wt/RHOA-G17V-pSB-bi-GP-G17V/empty-vector-pSB-bi-GP) and a 0.5 μg SB transposase construct (pCMV[CAT]T7-SB100, #34879, Addgene, Watertown, MA, USA). The cells were electroporated on a DF-110 programme using a 4D Nucleofector (Lonza, Basel, Switzerland). The selection of positive cells was performed using 1 µg/mL puromycin for 10–14 days and terminated when virtually all cells exhibited the expected green fluorescence and a transfection efficiency of >94% green fluorescent protein signal using FACS was confirmed. To confirm that the cell lines expressed the mutant or wild-type *RHOA* variant, RNA was extracted, and from the resulting cDNA, parts of the *RHOA* transcript were PCR-amplified and sequenced (Supplementary Figure S2).

### 2.2. Microchannel Experiments and 3D Collagen Gels

Polydimethylsiloxane (PDMS) chips with different types of microchannels were produced in moulds provided by Dr Matthieu Piel. Straight channels with a diameter of 8 µm and a height of 10 µm were tested and determined to be most appropriate for HH and HuT-78 cells. Cell motility was monitored using a phase-contrast microscope (Lumascope LS720, Etaluma, Carlsbad, CA, USA) under incubator conditions for 20 h, with time-lapse images taken every 10 min in straight channels. The cells in the videos from the microchannel experiments were segmented using a custom script. A 1.5-mg/mL collagen-cell mixture was prepared and added to 3D μ-slide chemotaxis chambers (Ibidi, Martinsried, Germany) in accordance with the manufacturer’s protocol https://ibidi.com/img/cms/products/labware/channel_slides/S_8032X_Chemotaxis/IN_8032X_Chemotaxis.pdf (accessed on 12 June 2023). The microscopic time-lapse observation was performed for 20 h using a Lumascope LS720 microscope (Etaluma). The cells’ track velocity, track distance, accumulated distance, Euclidean distance, track directness, and track speed were automatically analyzed by FastTrack AI from MetaVi Labs (https://metavilabs.com (accessed on 12 June 2023)).

### 2.3. Proteomics, Cleaved Collagen, and Matrix Metalloproteinase Assay

All cell lines underwent quantitative proteomics based on tandem mass tagging.

After embedding the cells in a 1.5-mg/mL collagen I matrix in an Ibidi µ-Slide Angiogenesis slide (Ibidi), the cells were allowed to move for 96 h under incubator conditions. The cleaved collagen was marked with an anti-Col1-3/4 C antibody (collagen type I cleavage site, Immunoglobe, Himmelstadt, Germany). For the analysis of matrix metalloproteinases (MMPs), the MMP array (ab134004, Abcam, Cambridge, UK) was applied.

### 2.4. Live Cell Imaging and Analysis of Collagen Fibrosis

Written informed consent was obtained from all patients in accordance with the Declaration of Helsinki, and the study was approved by the Institutional Review Board of the University Hospital Frankfurt (No 20-876aV). For live cell imaging, HH and HuT78 cells expressing *RHOA*-WT or *RHOA*-G17V were labelled with red and green cell trackers (CellTracker™ Deep Red and CMFDA, ThermoFisher, Waltham, MA, USA) and plated onto thick tissue slices of vital human lymphoid tissue. Endogenous B and T cells were labelled with Alexa Fluor 555-anti-human CD19 (polyclonal rabbit antibody; Bioss Antibodies, Woburn, MA, USA) and Alexa Fluor 647-anti-human CD3 (clone UCHT1; BD Biosciences, Franklin Lakes, NJ, USA), as previously described. The time-lapse movies were analyzed using Imaris Software version 9.9.1. Primary cases of AITL with and without the *RHOA*-G17V mutation were stained with a Masson’s Trichrome Stain Kit (Agilent, Santa Clara, CA, USA) and consequently digitalized using a Hamamatsu NanoZoomer S360 scanner (Hamamatsu, Shizuoka, Japan) at 40× magnification. The collagen content was automatically quantified using a FIJI/ImageJ1.53 pipeline (Supplementary Figure S3). The input sample was separated into two stains by applying color deconvolution. The required input color vectors were directly determined from the sample by selecting two regions of interest, and from the two separated channels, the final collagen binary image was computed.

## 3. Results

### 3.1. The Growth Behavior of the T-Cell Lymphoma Cell Lines HuT78 and HH Is Not Significantly Influenced by the Overexpression of RHOA-G17V

**Figure 1:**
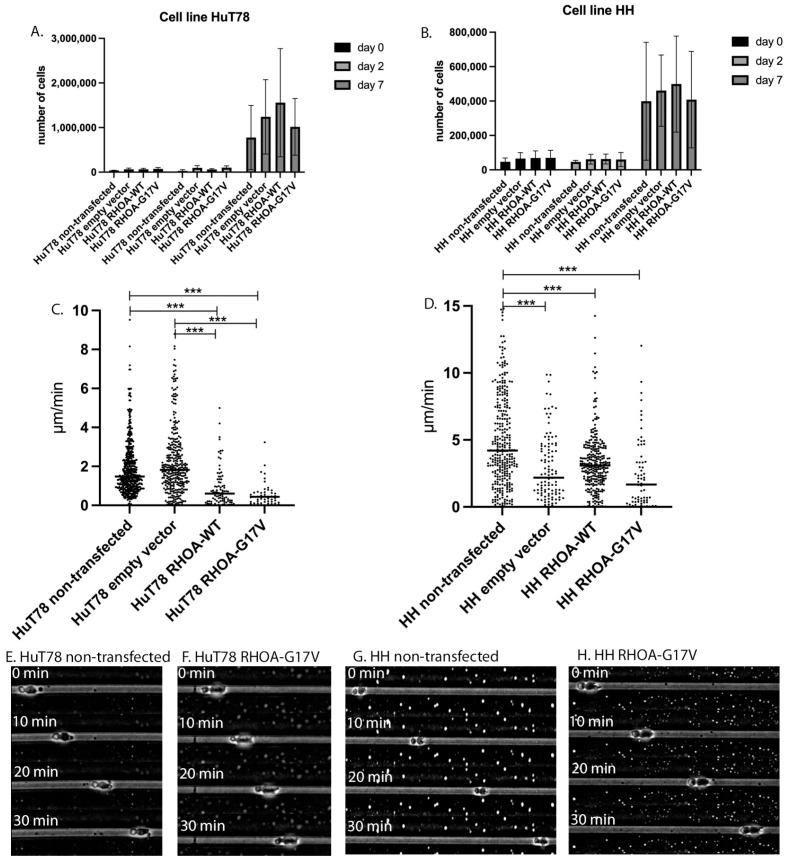
(**A**–**D**). Growth behavior and migration velocity in the PDMS microchannels of T-cell lymphoma cell lines expressing RHOA-G17V. (**A**) Growth behavior of non-transfected HuT78 cells, empty-vector-transfected HuT78 cells and RHOA-WT as well as RHOA-G17V-expressing HuT78 cells. Means and standard deviation from three independent experiments. (**B**) Growth curve of non-transfected HH cells, empty-vector-transfected HH cells and RHOA-WT as well as RHOA-G17V-expressing HH cells. Means and standard deviation from three independent experiments. (**C**) Velocity of non-transfected HuT78 cells, empty-vector-transfected HuT78 cells and RHOA-WT as well as RHOA-G17V-expressing HuT78 cells in PDMS microchannels with 10 µm height and 8 µm width. Each dot represents the mean velocity of one cell. Cells were measured in at least three independent experiments. *** *p* < 0.001, Kruskal–Wallis-test with Benjamini–Yekutieli post-test for multiple comparisons. (**D**) Velocity of non-transfected HH cells, empty-vector-transfected HH cells and RHOA-WT as well as RHOA-G17V-expressing HH cells in PDMS microchannels with 10 µm height and 8 µm width. Each dot represents the mean velocity of one cell. The cells were measured in at least three independent experiments. *** *p* < 0.001, Kruskal–Wallis-test with Dunn´s post-test for multiple comparisons. (**E**–**H**). (**E**) Example of a non-transfected HuT78 cell in a PDMS microchannel with uropod formation at the rear of the cell. (**F**) Example of a HuT78 cell expressing RHOA-G17V in a PDMS microchannel with uropod formation at the rear of the cell. (**G**) Example of a non-transfected HH cell in a PDMS microchannel with uropod formation at the rear of the cell. (**H**) Example of an HH cell expressing RHOA-G17V in a PDMS microchannel with uropod formation at the rear of the cell.

The cell lines Hut78 and HH transfected with *RHOA*-G17V, *RHOA*-WT, or an empty vector were seeded at approximately 5 × 10^4^ cells per well, and cell numbers were determined by FACS on day 0, day 2, and day 7, respectively. All cell lines had increased cell numbers after 7 days of culture without significant differences in cell numbers. The HuT78 cells were generally more proliferation-active than the HH cells. The cell lines transfected to express *RHOA*-WT had slightly higher cell numbers than those transfected to express *RHOA*-G17V (mean 4.99 × 10^5^ cells in HH *RHOA*-WT compared with 4.08 × 10^5^ in HH *RHOA*-G17V and mean 1.55 × 10^6^ cells in HuT78 *RHOA*-WT compared with mean 1.02 × 10^6^ in HuT78 *RHOA*-G17V, Figure 1A,B).

### 3.2. HH and Hut78 Cells Expressing RHOA-G17V Exhibited Impaired Motility in Straight Microchannels

To determine the impact of the *RHOA*-G17V mutation on motility, both HH and HuT78 cell lines were analyzed for their velocity in straight PDMS microchannels with a height of 10 µm and width of 8 µm, as previously described [26]. The non-transfected HH cells were generally faster than the non-transfected HuT78 cells (mean 5.13 µm/min vs. 1.90 µm/min). The HuT78 cells expressing *RHOA*-G17V moved significantly slower than the non-transfected HuT78 cells and the HuT78 cells transfected with an empty vector (means of 0.57 µm/min vs. 1.90 µm/min (untreated) vs. 2.27 µm/min (empty vector), *p* < 0.05, Kruskal–Wallis test with Dunn’s post-test for multiple comparisons, Figure 1C). The HuT78 cells transfected to overexpress *RHOA*-WT also moved at a lower velocity (mean 1.12 µm/min), and the *RHOA*-G17V-expressing HuT78 cells less frequently entered the microchannels than the non-transfected cells (49 *RHOA*-G17V-expressing HuT78 cells compared with 519 non-transfected HuT78 cells, sum of three independent experiments). The *RHOA*-G17V-expressing HH cells exhibited similarly reduced velocity compared with the non-transfected HH cells (mean velocity 2.43 µm/min in *RHOA*-G17V HH cells vs. 5.13 µm in non-transfected HH cells, *p* < 0.001, Kruskal–Wallis test with Dunn’s post-test for multiple comparisons, Figure 1D). Similar to HuT78, the HH cells expressing *RHOA*-G17V less frequently entered the PDMS microchannels compared with the non-transfected HuT78 cells (67 *RHOA*-G17V-expressing cells vs. 309 untreated HH cells, sum of three independent experiments). In contrast with the reduced motility observed in the HuT78 and HH cells expressing *RHOA*-G17V, the morphology of the moving cells was comparable to that of the non-transfected cells (Figure 1E–H), with a uropod at the rear of the cells, which is usually observed in moving T cells [27].

### 3.3. HuT78 and HH Cells Expressing RHOA-G17V Do Not Differ in Their Movement Patterns in 3D Collagen Gels and Human Lymphoid Tissue

Because we observed the significantly reduced motility of *RHOA*-G17V-expressing T-cell lymphoma cells in the PDMS microchannels, we were interested to investigate the effects of *RHOA*-G17V in a more physiological environment, such as a 3D collagen gel and an in-vivo-like slice model of living human lymphoid tissue. First, the HuT78 and HH cells were ingested in a 3D collagen gel, and the velocity of the cells, accumulated track length, and distance of displacement were evaluated, as described previously [28]. In both cell lines, the mean velocity in the 3D collagen gel (0.15 µm/min for the non-transfected HH cells and 0.18 µm/min for the non-transfected HuT78 cells) was much slower than in the PDMS microchannels. However, overall, there was no significant difference in velocity, nor the accumulated track or distance of displacement between the HH and HuT78 cells transfected with an empty vector or overexpressing *RHOA*-WT or *RHOA*-G17V (Supplementary Figure S4). The velocity of the HH and HuT78 cells overexpressing *RHOA*-G17V was slightly reduced in the 3D collagen gel (0.14 µm/min for HH *RHOA*-G17V and 0.12 µm/min for HuT78 *RHOA*-G17V) when compared with the empty-vector-transfected cells (0.20 µm/min for the empty-vector-transfected HH cells and 0.19 µm/min for the empty-vector-transfected HuT78 cells).

**Figure 2:**
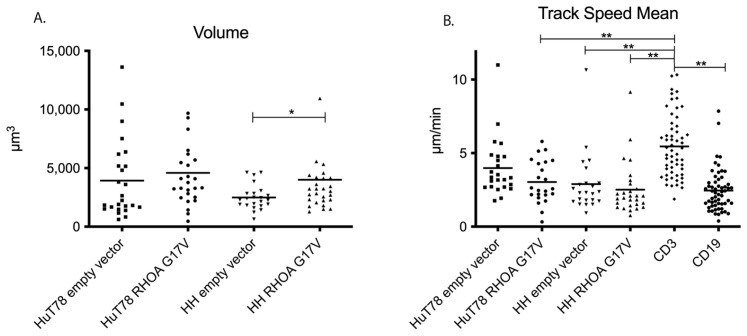

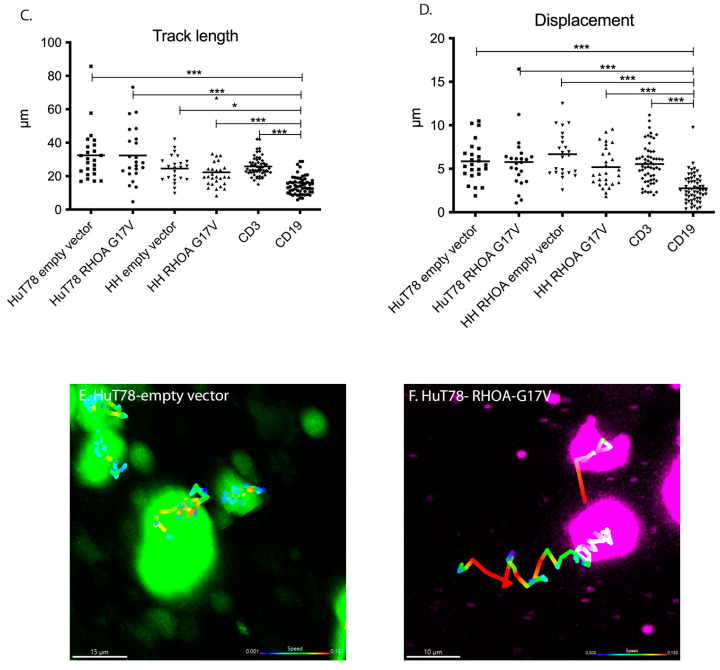
(**A**,**B**) Volume, track speed, track length, and displacement of T-cell lymphoma cell lines expressing RHOA-G17V in native lymphoid tissue. (**A**) Volume of cell bodies (means). Each dot represents the volume of one cell from three independent experiments. * *p* = 0.039, Mann–Whitney-test. (**B**) Mean track speed of the empty-vector-transfected or RHOA-G17V-overexpressing HuT78 and HH cells compared with the endogenous human-CD3-positive T cells and the CD19-positive B cells. Each dot represents the mean track-based speed of one cell; the cells were recorded in three independent experiments. ** *p* < 0.01, Kruskal–Wallis test with Dunn´s post-test for multiple comparisons. (**C**) Track length of the empty-vector-transfected or RHOA-G17V-overexpressing HuT78 and HH cells compared with endogenous human-CD3-positive T cells and the CD19-positive B cells. Each dot represents the track length of one cell; the cells were recorded in three independent experiments. * *p* < 0.05, *** *p* < 0.001, Kruskal–Wallis test with Dunn´s post-test for multiple comparisons. (**D**) Displacement of the empty-vector-transfected or RHOA-G17V-overexpressing HuT78 and HH cells compared with the endogenous human-CD3-positive T cells and CD19-positive B cells. Each dot represents the track length of one cell; the cells were recorded in three independent experiments. *** *p* < 0.001, Kruskal–Wallis test with Dunn´s post-test for multiple comparisons. (**E**) Examples of empty-vector-transfected HuT78 cells (green) with reconstructed tracks of a 15 min movie in native lymphoid tissue. (**F**) Examples of RHOA-G17V-expressing HuT78 cells (pink) with reconstructed tracks of a 15 min movie in native lymphoid tissue.

The motility of the *RHOA*-G17V-expressing cells and the empty-vector-transfected cells was then evaluated in primary lymphatic tissue. The cell volume was significantly higher in the HH *RHOA*-G17V cells compared with the empty-vector-transfected HH cells (mean 4009 µm^3^ vs. 2490 µm^3^, *p* = 0.04, Mann–Whitney test). The HuT78 cells expressing *RHOA*-G17V had only slightly higher cell volumes when compared with the already relatively large empty-vector-transfected cells (*RHOA*-GI7V: mean 4594 µm^3^; empty vector: 3933 µm^3^; not significant, Figure 2A). The velocity of both cell lines expressing *RHOA*-G17V was only slightly reduced when compared to the cells transfected with an empty vector (HuT78 *RHOA*-G17V: 3.03 µm/min; empty vector HuT78: 3.99 µm/min; not significant; HH *RHOA*-G17V: 2.51 µm/min empty vector HH: 2.90 µm/min; not significant, Figure 2B). All cells derived from T-cell lymphoma cell lines were slower compared with the endogenous human T cells identified by CD3 staining (endogenous T cells: mean velocity of 5.46 µm/min, Figure 2B). The difference between the tissue-resident endogenous T cells and both HuT78 and HH cells transfected to express *RHOA*-G17V and the empty-vector-transfected HH cells was significant (*p* < 0.01, Kruskal–Wallis test with Dunn’s post-test for multiple comparisons). Surprisingly, despite the overall observed reduced velocity of HuT78 and HH cells expressing *RHOA*-G17V, both the track length and displacement were not significantly different between the HH and HuT78 cells and the tissue-resident endogenous T cells (Figure 2C–F). As we hypothesized that the *RHOA*-G17V mutation may alter the interactions with bystander cells, the numbers and duration of cell–cell contacts between the lymphoma cell lines HH and HuT78 expressing RHOA-G17V or transfected with an empty vector and the CD19- and CD3-positive endogenous human lymphoid cells were measured; however, there was no difference between the HH and HuT78 cells expressing *RHOA*-G17V and *RHOA*-WT (Supplementary Figure S5).

### 3.4. Global Proteomics Screening Reveals Decreased Expression of RHOG and RHOF in HuT78 and HH Cells Expressing RHOA-G17V

As we generally observed reduced motility in the HuT78 and HH cells expressing *RHOA*-G17V, global proteomics screening of the T-cell lymphoma cell lines expressing *RHOA*-G17V was performed in triplicate and compared with the empty-vector-transfected cells. The resulting differences in protein expression were generally mild. Four genes were commonly upregulated (>1.1-fold) in both cell lines (HuT78 and HH) expressing *RHOA*-G17V (Table 1) compared with the empty-vector-transfected cells in addition to 14 commonly downregulated proteins (Table 2), which surprisingly included two other small GTPases, *RHOF* and *RHOG*.

### 3.5. Collagen Cleavage Is Observed in HH Cells Expressing RHOA-G17V and Reduced Collagen Content Is Found in AITL Patients with High RHOA-G17V Allele Frequencies

**Figure 3:**
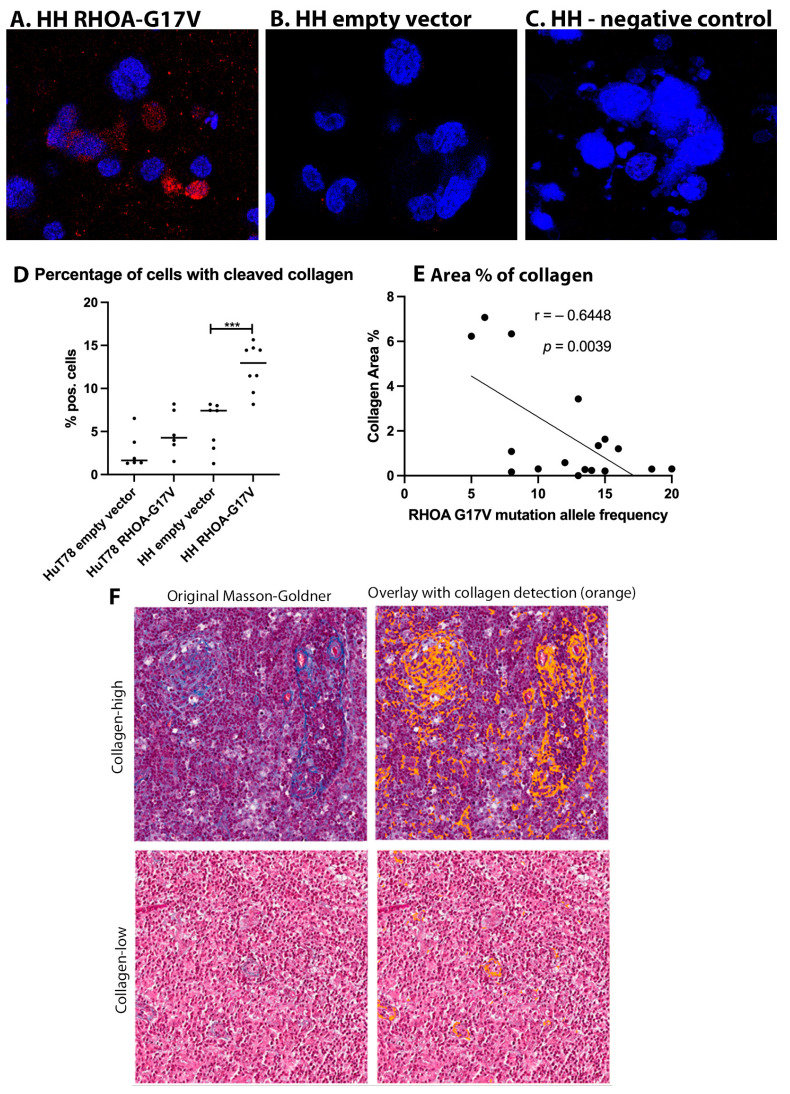
Collagen cleavage around HuT78 and HH cells and collagen content in primary AITL cases. (**A**) Cleaved collagen (red) around the RHOA-G17V-expressing HH cells (63× magnification). (**B**) Cleaved collagen (red) around the HH cells transfected with the empty vector (63× magnification). (**C**) Negative control: HH cells expressing RHOA-G17V stained without the primary antibody against cleaved collagen (63× magnification). (**D**) Percentage of cells with cleaved collagen in the empty-vector-transfected HuT78 and HH cells or expressing RHOA-G17V. Each dot corresponds to the mean percentage in a gel image. *** *p* < 0.001, Mann-Whitney-test. (**E**) Percentage of area occupied by collagen in histologic slices of lymph nodes infiltrated by AITL in relation to the allele frequency of RHOA-G17V mutation. (**F**) Examples of two AITL cases with high (upper panels, *RHOA-G17V* allele frequency 8%) and low (lower panels, *RHOA-G17V* allele frequency 12%) collagen content. Left: original Masson–Goldner stains; right: overlay with collagen content (orange).

Because we observed reduced velocity of T-cell lymphoma cell lines expressing *RHOA*-G17V, we aimed to determine whether a switch occurs from amoeboid to mesenchymal migration, characterized by the production of MMPs by the migrating cells. First, supernatants and cell lysates of the *RHOA*-G17V-expressing HH and HuT78 cells were analyzed with an MMP assay detecting MMP1, 2, 3, 8, 9, 10, and 13. However, no relevant expression or secretion of the respective MMPs was detected in the cell lysates or supernatants (Supplementary Figure S6). Next, the *RHOA*-G17V-expressing and empty-vector-transfected cells were embedded in a 1.5 mg/mL collagen I gel, and after an incubation period of 96 h, cleaved collagen was detected using an antibody against cleaved collagen I (cleavage site Col1–3/4). Only a few cells were detected that exhibited cleavage of collagen I (Figure 3A–D, means: 2.71% in HuT78 empty vector, 4.87% in HuT78 *RHOA*-G17V, 5.62% in HH empty vector and 12.49% in HH *RHOA*-G17V). However, the number of cells with cleaved collagen in HH *RHOA*-G17V was significantly higher than in the empty-vector-transfected HH cells (*p* < 0.001, Mann–Whitney test).

To understand whether the degradation of collagen fibres contributes to the dissemination of AITL in patients, primary cases of AITL were evaluated for their collagen density on histological sections. Collagen density (% area) was significantly negatively correlated with the allele frequency of the *RHOA*-G17V mutation in the tissue (Figure 3E,F, r = −0.6448, *p* = 0.0039). However, when the AITL cases with a *RHOA*-G17V mutation were compared with the *RHOA*-WT AITL cases, the difference in collagen density was moderate (median of 0.30% in *RHOA*-G17V AITL and 1.69% in *RHOA*-WT AITL, not significant, Supplementary Figure S7).

## 4. Discussion

Most AITLs harbor the *RHOA* mutation G17V; however, the effect of this mutation on the specific presentation of AITL remains unclear.

Because AITL has a characteristic clinicopathological presentation with early dissemination in the human body and additionally spreads to perinodal tissues, we hypothesized that the neoplastic cells in AITL would acquire enhanced motility via the *RHOA*-G17V mutation. In contrast with our expectations, in the PDMS microchannels, 3D collagen gels, and primary human lymphoid tissue, we observed a reduced or unchanged velocity of HH and HuT78 cells expressing *RHOA*-G17V. Despite the slightly reduced mean velocities of these cells, track length and cell displacement were not significantly altered, indicating that HH and HuT78 cells expressing *RHOA*-G17V are still capable of dissemination. Surprisingly, in the cells expressing *RHOA*-G17V, other small GTPases, such as *RHOF* and *RHOG*, were downregulated. Like other *RHO*-encoded proteins, *RHOG* promotes the reorganization of the actin cytoskeleton and regulates cell shape, attachment, and motility. The encoded protein facilitates the translocation of a functional guanine nucleotide exchange factor (GEF) complex from the cytoplasm to the plasma membrane, where ras-related C3 botulinum toxin substrate 1 is activated to promote lamellipodium formation and cell migration. In *RHOG*-knockdown HeLa cells, activation of Rac1 and the formation of lamellipodia at the leading edge were attenuated in response to wounding [29]. Therefore, the downregulation of *RHOF* and *RHOG* in addition to the inactivation of *RHOA* by the mutation G17V may hamper the migration capacity of *RHOA*-G17V-expressing cells. However, as RHOF and *RHOG* are considered typical Rho GTPases, their activity is not exclusively regulated by expression levels but also by conformation changes due to the binding of GTP [30].

Furthermore, we observed increased collagen cleavage in the HH cells expressing *RHOA*-G17V when compared with the empty-vector-transfected cells. Importantly, when the primary AITL patient samples were assessed, a negative correlation between collagen fibrosis and the allele frequency of the *RHOA*-G17V mutation was observed, suggesting degradation of the extracellular matrix by lymphoma cells, which may represent an alternative mechanism for enhancing the dissemination of the lymphoma cells. As we did not demonstrate the degradation of the extracellular matrix by the *RHOA*-G17V-expressing lymphoma cells, different mechanisms may lead to reduced collagen content, which may be the result of a lack of collagen synthesis in these cases or collagen degradation by bystander cells. In cytotoxic T cells, it has been described that these can form channels in a collagen matrix [31]. In solid tumors, degradation of collagen in the extracellular matrix was mainly the result of MMP secretion by fibroblasts [32]. Interestingly, we could not demonstrate enhanced MMP secretion in the T-cell lymphoma cell lines expressing *RHOA*-G17V; however, we cannot rule out that *RHOA*-G17V-expressing T cells can stimulate fibroblasts or other cells in the microenvironment to secrete MMPs and thus contribute to the remodelling of the extracellular matrix. Remodelling of the extracellular matrix characterized by synthesis and degradation is a process that occurs in a variety of physiological and pathological conditions [33] including in chronic inflammation and autoimmune diseases. Cleavage of some proteins of the extracellular matrix can result in structures similar to chemokines and cytokines that can promote cell migration and inflammatory signalling [34]. Cleavage of extracellular matrix components may therefore have an impact on the migration of lymphoma cells which is beyond that of a mechanical barrier.

One major challenge and limitation in understanding AITL is the lack of representative cell lines that can be studied. This is related to the fact that AITL is a rare tumor and that the tumor microenvironment, which is lacking in cell cultures, contributes to the development of this tumor. Therefore, as a model system, we chose two human CD4^+^ cutaneous T-cell lymphoma cell lines that harbored mutations in *TP53* (http://cellosaurus.org (accessed on 12 June 2023)) [35], which is frequently seen in cutaneous T-cell lymphomas [21], and both were *RHOA*-WT (according to depmap: Cancer Dependency Map, Broad Institute, 415 Main Street, Cambridge, MA 02142, USA and own Sanger sequencing of codon 17). HuT78 has an additional *NRAS*-Q61K mutation [36]. According to depmap, HuT78 harbors a stop mutation (Q608*) of *TET3*, which is a paralog of *TET2*, which is frequently mutated in AITL [37]. Furthermore, some primary cutaneous T-cell lymphomas harbor *RHOA* mutations, usually the N117I mutation [21]. Although both cell lines (HuT78 and HH) derived from CD4^+^ cutaneous T-cell lymphomas are probably similar to normal CD4^+^ T cells, they lack mutations in *TET2*, *DNMT3A*, and *IDH2* in contrast with the neoplastic cells of AITL. Therefore, in the present study, we report on the isolated effects of the *RHOA*-G17V mutation in neoplastic CD4^+^ T cells. We did not observe the proliferative advantage for the cells expressing *RHOA*-G17V that other authors reported when overexpressing *RHOA*-G17V in normal T cells [19] or T-cell lymphoma [13] due to the fact that we analyzed neoplastic cells with a *TP53* aberration.

## 5. Conclusions

In summary, our data indicate that the typical presentation of AITL with disseminated disease is probably related to factors such as remodelling of the extracellular matrix and it is not the result of improved migration efficacy of the tumor cells despite a mutation in the RHOA protein, well known for its role in cell motility.

## Figures and Tables

**Table 1:** List of proteins overexpressed in HH and HuT78 cells expressing RHOA-G17V compared with the empty-vector-transfected cells.

Gene Symbol	HH-RHOA G17V/Empty Vector Ratio	HuT78-RHOA G17V/Empty Vector Ratio	Mean Ratio	Name
ANXA4	1.27	1.13	1.20	Annexin A4
ASS1	1.14	1.16	1.15	Argininosuccinate Synthase 1
CDC26	1.13	1.13	1.13	Cell Division Cycle 26
RFFL	1.19	1.39	1.29	Ring Finger And FYVE-Like Domain Containing E3 Ubiquitin Protein Ligase

Given are the fold changes of protein expression of the means of three independent experiments each.

**Table 2:** List of proteins with reduced expression in HH and HuT78 cells expressing RHOA-G17V compared with the empty-vector-transfected cells.

Gene Symbol	HH-RHOA G17V/Empty Vector Ratio	HuT78-RHOA G17V/Empty Vector Ratio	Mean Ratio	Name
ZNF770	0.65	0.80	0.72	Zinc Finger Protein 770
**RHOF**	**0.81**	**0.68**	**0.75**	**Ras Homolog Family Member F, Filopodia Associated**
BRPF1	0.84	0.69	0.77	Bromodomain and PHD Finger Containing 1
PECR	0.73	0.83	0.78	Peroxisomal Trans-2-Enoyl-CoA Reductase
KAT5	0.72	0.86	0.79	Lysine Acetyltransferase 5
SLC43A3	0.72	0.88	0.80	Solute Carrier Family 43 Member 3
**RHOG**	**0.88**	**0.74**	**0.81**	**Ras Homolog Family Member G**
MED19	0.83	0.82	0.82	Mediator Complex Subunit 19
RELCH	0.85	0.83	0.84	RAB11 Binding and LisH Domain, Coiled-Coil and HEAT Repeat Containing
RETSAT	0.88	0.87	0.88	Retinol Saturase
CAV1	0.88	0.88	0.88	Caveolin 1
PRNP	0.88	0.89	0.88	Prion Protein
TYMS	0.89	0.88	0.89	Thymidylate Synthetase
DDX10	0.90	0.88	0.89	DEAD-Box Helicase 10

Given are the fold changes of protein expression of the means of three independent experiments each. Small GTPases involved in cell motility are highlighted in bold.

## Data Availability

Data are available from the corresponding author upon reasonable request.

## Supplementary Materials

The following supporting information can be downloaded at: https://www.mdpi.com/article/10.3390/cancers15123226/s1. Figure S1. Typical example of a lymph node involved by angioimmunoblastic T-cell lymphoma. A. Infiltrates of small to medium-sized T-cells expand into the surrounding perinodal fatty tissue, leaving open the marginal sinus (HE, 7×). B. CD3 immunostaining highlights that the majority of the infiltrate are T cells, expanding to perinodal fatty tissue (5×). C. PD1 immunostaining highlights that the neoplastic cells expand to the perinodal tissue (6×). D. Higher magnification of PD1 immunostaining (10×) shows infiltration of neoplastic cells into perinodal tissue. Figure S2. Sanger Sequencing of cDNA obtained from RNA from HH and HuT78 cells transfected to stably express RHOA-G17v, RHOA-WT or empty vector transfected cells. Figure S3. Fiji pipeline for the automated quantification of collagen in tissue slices. A. Selection of a representative area within a lymph node section. B. Procedure of the Fiji pipeline applying first a colour deconvolution, conversion to a binary image using a specific threshold, substraction and filtering. Figure S4. Velocity, track length and displacement in a 3D collagen gel of HuT78 and HH cells untreated, empty vector transfected or transfected with vectors to stably express RHOA-WT or RHOA-G17V. Figure S5. Analysis of cell-cell contacts of RHOA-WT and RHOA-G17V expressing HuT78 and HH cells with endogenous tissue-resident B- (CD19) and T- (CD3) cells A. and B. Quantification of the number of contacts (mean number of contacts/movie) C. and D. Mean duration of contacts per movie. Figure S6. MMP assay of HuT78 and HH cell lysates of empty vector transfected or RHOA-G17V expressing cells or supernatants collected from RHOA-G17V expressing cells. The sarcoma cell line HT-1080 was used as positive control. Figure S7. Automatically quantified collagen content (area %) in AITL samples with and without RHOA-G17V mutation.
