# Supplementary material for: The RHOA Mutation G17V Does Not Lead to Increased Migration of Human Malignant T Cells but Is Associated with Matrix Remodelling
Source: Cancers (Basel). 2023 Jun 17;15(12):3226. doi: 10.3390/cancers15123226 (PMC10296629; doi:10.3390/cancers15123226)
Supplement: Supplementary file 1 [file cancers-15-03226-s001.zip › cancers-2445343-supplementary.pdf]

Supplementary Methods and Data

Supplementary Methods

Cell culture, Cloning and Transfection

Cell culture

The $CD4^+$ cutaneous T-cell lymphoma cell line HH (CVCL_1280, https://www.cellosaurus.org), was obtained from German Collection of Microorganisms and Cell Cultures (Braunschweig, Germany) and was maintained in complete cell culture medium (RPMI) with 10 % fetal bovine serum (FBS). HuT78, (CVCL_0337, https://www.cellosaurus.org) another CD4+ cutaneous T-cell lymphoma cell line (Sézary lymphoma), was kindly provided by PD Dr. Marco Herling, University of Leipzig, and maintained in IMDM complete medium supplemented with 20% FBS. The identity of both cell lines was authenticated by short tandem repeats (STR) profiling. All cell lines were regularly tested for mycoplasma contamination.

Cloning

For the generation of T-cell lymphoma cell lines stably expressing RHOA-WT and RHOA-G17V, the respective sequences were cloned into a sleeping beauty vector pSBbi-GP by using the sleeping beauty system as described by Kowarz et al. [1]. For the human RHOA-WT sequence, the plasmid pcDNA-EGFP-RhoA-WT (#12965, Addgene, Watertown, MA, USA) was purchased. The RHOA-WT sequence was amplified from the plasmid pcDNA-EGFP-RHOA using specific primers containing the SfiI restriction site. The mutated form of human RHOA-G17V (c.50G>T Gly17Val) was generated using the site directed mutagenesis kit (Quikchange, Agilent, Santa Clara, CA, USA). Further, the vector of the transposon sleeping beauty system pSBbi-GP (#60511, Addgene) and the amplified RHOA insert with and without RHOA-G17V were digested using SfiI enzyme. Ligation of the vector and insert were performed using the Rapid Ligation Kit (Thermofisher, Waltham, MA, USA) and XL10-Gold ultracompetent cells (Agilent) were used for transformation. After screening for positive clones and plasmid DNA purification, the resulting constructs pSBbi-GP-RHOA-WT and pSBbi-GP-RHOA-G17V were confirmed by Sanger sequencing.

Nucleofection of HH and HuT78 cells

2 × 10^6 cells per sample were co-transfected with 5 µg/100µL SB transposon carrying DNA plasmids (RHOA-WT-pSB-bi-GP-wt/ RHOA-G17V-pSB-bi-GP-G17V/ empty-vector-pSB-bi-GP) and 0,5 µg SB transposase construct (pCMV(CAT)T7-SB100, #34879, Addgene). In some control experiments, cells were nucleofected with 0,5 µg pCMV(CAT)T7-SB100 alone or were subjected to nucleofection without DNA. Cells were electroporated on DF-110 program of a 4D Nucleofector (Lonza, Basel, Switzerland). After 48 h later, appropriate selection was carried out with 1 µg/mL puromycin. Selection was carried out for 10 to 14 days and terminated when virtually all cells showed the expected green fluorescence and sufficient transfection efficiency of > 94 % GFP signal using FACS. In order to confirm that the cell line express the mutant or wild type RHOA variant, RNA was extracted using the RNeasy Mini Kit (Qiagen, Hilden, Germany) and cDNA was transcribed using the High Capacity cDNA Reverse Transcription Kit (#4368814, Applied Biosystems) using random hexamers. From the resulting cDNA, the RHOA transcript was PCR amplified and expression of the RHOA-G17V mutant or RHOA-WT was confirmed in the respective cell lines (Suppl. Figure S1).

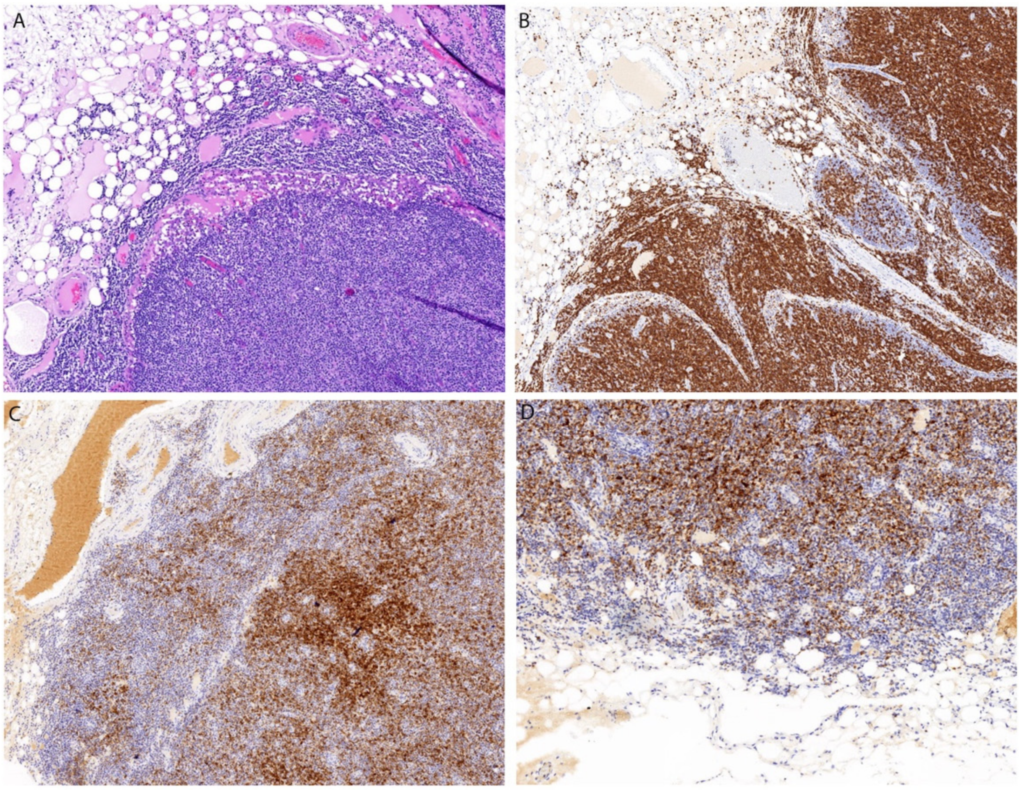

Suppl. Figure S1. Typical example of a lymph node involved by angioimmunoblastic T-cell lymphoma.

A. Infiltrates of small to medium-sized T-cells expand into the surrounding perinodal fatty tissue, leaving open the marginal sinus (HE, 7x). B. CD3 immunostaining highlights that the majority of the infiltrate are T cells, expanding to perinodal fatty tissue (5x). C. PD1 immunostaining highlights that the neoplastic cells expand to the perinodal tissue (6x). D. Higher magnification of PD1 immunostaining (10x) shows infiltration of neoplastic cells into perinodal tissue.

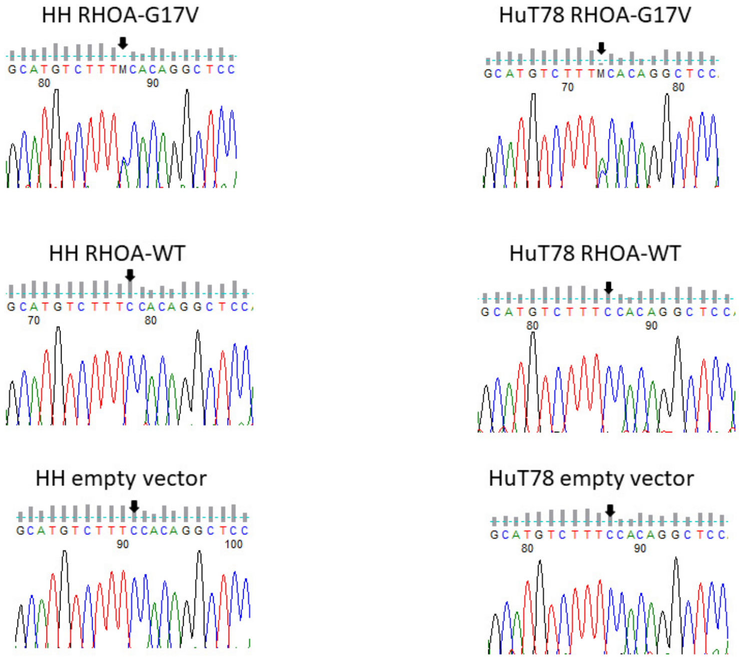

Suppl. Figure S2. Sanger Sequencing of cDNA obtained from RNA from HH and HuT78 cells transfected to stably express RHOA-G17v, RHOA-WT or empty vector transfected cells

Microchannel experiments

Polydimethylsiloxane (PDMS) chips with different types of microchannels were produced in molds provided by Dr. Matthieu Piel. Straight channels with a diameter of 8 μm and height of 10 μm were tested and found to be most appropriate for HH and HuT78 cells. Microchannels were coated with fibronectin for at least 1 hour and then 10^6 cells were loaded into the PDMS chip, and subsequently pre-incubated for 1 h at 37°C in the presence of 5% of CO2. Cell motility was monitored using a phase-contrast microscope (Lumascope LS720, Etaluma, Carlsbad, CA, USA) under incubator conditions for 20 h, with time-lapse images taken every 10 min in straight channels.

Cells in videos from microchannel experiments were segmented with a custom script (available on request; see below for a description of the procedure). The X and Y positions of the segmented cells were used as input for tracking, which was done with TrackMate using the LAP tracker (parameters: maximum distance for frame-to-frame linking: 100; maximum distance for gap closing: 150; maximal gap length: 15; penalty for linking cells with different Y positions: Y15). The step-based velocity and straightness were calculated based on the X, Y, and T coordinates of the tracks.

Segmentation of movies from microchannel experiments

Segmentation was done with the scikit-image package in Python [2]. First, noise was removed with a median filter, and a background image was calculated by taking the median pixel value for each position over all frames of the video. Then, this background image was subtracted from each frame to remove the microchannels from the image. A mean filter was applied to smoothen the image again and a black top-head filter was used to enhance the signal of the cells. Finally, the cells were detected with a Yen threshold, and morphological operations were used to improve the result.

3D collagen gels

A collagen-cell mixture was prepared and filled into 3D μ-slide chemotaxis chambers (Ibidi, Martinsried, Germany) in accordance with the manufacturer's protocol. The μ–Slide Chemotaxis was provided with a Collagen coated surface and 9 x 10^5 cells of either HuT78 or HH cells were used per chamber in 50 μl volume. Microscopic time lapse observation was carried out for 20 hours with a Lumascope LS720 microscope (Etaluma) at 10 × magnification at incubator conditions Every 10 min a bright field picture with a microscopy resolution of 1,19 pixel/μm was generated. The cells' track

velocity, track distance, accumulated distance, euclidean distance, track directness as well as track speed of HH and HuT78 cells with RHOA mutation, WT and empty vector were analyzed automatically by FastTrack AI from MetaVi Labs (https://metavilabs.com).

Proteomics

All cell lines underwent quantitative proteomics based on tandem mass tagging (TMT). For this, 5 × 10^6 cells were harvested and lysed in urea buffer as previously described. After protein extraction and digestion with trypsin, peptides were labeled with TMT 10-plex reagents (Thermo Fisher Scientific, Dreieich, Germany). The individually labeled peptides were combined in multiplexed samples. For normalization among multiplexes, an internal reference consisting of peptides from each condition was included. After pre-fractionation using a high-pH C18 reversed-phase kit (Thermo Fisher Scientific), the TMT-labeled peptide mixtures were separated by C18-reversed-phase-HPLC on an Ultimate 3000 RSLCnano system (Thermo Fisher Scientific). Peptides eluted from the analytical column were analyzed by nano-ESI Orbitrap tandem mass spectrometry on a Q Exactive HF instrument (Thermo Fisher Scientific). The raw mass spectrometric data were processed with the MaxQuant software (version 1.6.17.0; Max Planck Institute of Biochemistry [MPIB]). This included the identification of proteins by running the mass spectra against the Uniprot human protein database (downloaded in February 2019) and collection of common lab contaminants, followed by the extraction of TMT reporter ion intensities for protein quantitation. After recalibration, the mass tolerances for precursor and fragment ions were set to 4.5 ppm and 20 ppm, respectively. Oxidation of methionine and acetylation of the protein *N*-terminus were considered variable modifications, and carbamidomethylation of cysteine was defined as a fixed modification. The minimal peptide length was set to seven amino acids, allowing up to two missed tryptic cleavages. Both at the peptide and protein levels, a maximum false discovery rate (FDR) of 1% was applied using a reversed decoy database. For downstream data processing with the Perseus software (version 1.6.0.7; MPIB), potential contaminants, proteins identified solely with modified peptides, and hits in the decoy database were removed. After normalization of TMT reporter ion intensities across multiplexed samples, the data were subjected to unsupervised hierarchical clustering based on the Euclidean distance and average linkage method, either using all quantified protein groups or after mapping motility-related proteins.

Cleaved Collagen and MMP Assay

After embedding of cells in a 1.5 mg/ml collagen I matrix in an Ibidi µ-Slide Angiogenesis slide (Ibidi #81506), cells were allowed to move for 96 hours under incubator conditions. Fixation was performed with 4% paraformaldehyde. Degraded collagen was marked with anti-Col1-3/4 C (collagen type I cleavage site) antibody (1:100) and dectected by the VectaFluor™ DyLight 594 Kit (VectaFluor™ Excel, Vector Laboratories, Newark, CA, USA). Cell nuclei were visualized with DAPI (1:500). Images were acquired with a Leica SP8 confocal microscope. Cell nuclei were automatically counted in Fiji. The red signal of cleaved collagen was manually counted by two independent investigators (K.M.A. and S.H.) and the mean percentage of positive cells was taken for further analysis.

For the analysis of matrix metalloproteinases (MMPs) the MMP array (ab134004, Abcam) was applied. Lysates and supernatants from RHOA-G17V expressing and empty vector transfected cells were collected and subjected onto the array. As positive control, lysates of the sarcoma cell line HT1080 were used.

Live cell imaging

For live cell imaging, HH and HuT78 cells expressing RHOA-WT or RHOA-G17V were labelled with red and green cell trackers (CellTracker™ Deep Red, #C34565, ThermoFisher CellTracker™ CMFCA, #C2925, ThermoFisher) and plated onto 350 µm thick vital tissue slices of human lymphoid tissue obtained from routine adenectomy samples of five individuals. Written informed consent was obtained from all patients in accordance with the Declaration of Helsinki and the study was approved by the Institutional Review Board of the University Hospital Frankfurt (No 20-876aV). After an incubation time of 30 min under incubator conditions, time-lapse movies were recorded with a Leica SP8 confocal microscope (Leica Microsystems) under incubator conditions every 15 sec for a period of 15 min. Endogenous B and T cells were labelled with Alexa Fluor 555-anti-human CD19 (polyclonal rabbit antibody; Bioss Antibodies, Woburn, MA, USA) and Alexa Fluor 647–anti-human CD3 (clone UCHT1; BD Biosciences, Franklin Lakes, NJ, USA) as previously described. Time-lapse movies were analysis using Imaris Software.

Data Analysis

Cellular parameters were calculated after three-dimensional reconstruction of the sequential z series using Imaris software. Automated tracking of the respective cells was done by Imaris Spot Detection. The "Estimated Diameter" settings were 8 µm for CD3-positive and for CD19-positive cells, and 12 µm for HuT78 and HH cells. The filter threshold was adjusted by visual inspection. Velocities were calculated according to the tracking tool "Autoregressive Motion." The settings were "MaxDistance" on the same value as the "Estimated Diameter", "MaxGapSize" 3, and "Fill Gap Enable" true. Calculation of cell–cell contacts assessed the number of cells closer than a defined distance to the center of another cell. According to the surface staining, first, the diameter of all CD3-positive, CD19-positive cells and HuT78 and HH cells was calculated in three dimensions. For the detection of a cell-cell contact "Classify Spots" of Imaris was used. HuT78 and HH cells were classified by "Shortest Distance to Spot", as a CD3- or CD19-positive cell, respectively. For cell-cell contacts all cells with a distance of their centers less than 12 µm were taken into account. The value was estimated by visual inspection. For statistical analyses, Gaussian distribution was tested using the Kolmogorov–Smirnov test. For two-group comparisons, either an unpaired t-test or the Mann–Whitney test was performed. Comparison of more than two groups used a one-way analysis of variance (ANOVA) with Bonferroni's post-test for multiple comparisons or a Kruskal–Wallis test with Dunn's post-test for multiple comparisons.

Analysis of collagen fibrosis

Primary cases of AITL with (n=18) and without RHOA G17V (n=21) mutation were stained with the Masson´s Trichrome Stain Kit (Agilent) and consequently digitalized by a Hamamatsu NanoZoomer S360 scanner (Hamamatsu, Shizuoka, Japan) in 40x magnification. Mutation analysis was performed in diagnostic standard with pyrosequencing using 5´-ACTTCTTGTGCATTGCAGGTAATA and 5´-TCTCAAACACTGTGGGCACATA primers for amplification of the fragment and the 5´-TTGGTGATGGAGCCT primer for sequence analysis. To quantify the amount of collagen automatically, a computer vision pipeline was established. An overview of the pipeline is depicted in Suppl. Figure S2. The pipeline was created in FIJI/ImageJ1.53 and consisted of standard functions of ImageJ. From the whole slide image, a large region was manually selected to be analysed (Suppl. Figure S2). This circumvented that extranodal connective tissue or large sinus structures were included in the

analysis. The input sample was separated into the two stains by applying a colour deconvolution. The required input colour vectors were directly computed from the sample by selecting two regions of interest. The two separated channels were then converted to a binary image using a threshold. The thresholds were set to ~200 for chanel 1 (blue) and less strict, ~100, for channel 2 (magenta). The ‚Image calculator‘ tool was selected to subtract the channel 2 binary from the channel 1 binary. The ‚Analyze particles‘ tool allows the identification of small particles (< 40 pixels), which were excluded from the result. The final image is 1 (collagen positive) in all regions where the original image has high values of the blue color, but low values in the red color vector. The relative amount of collagen positive pixels were computed with the ‚Measure‘ tool of Fiji. The correlation between RHOA G17V allele frequency and area % collagen fibrosis was calculated by a simple linear regression.

A

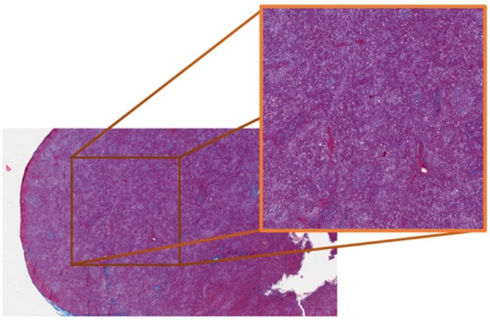

B

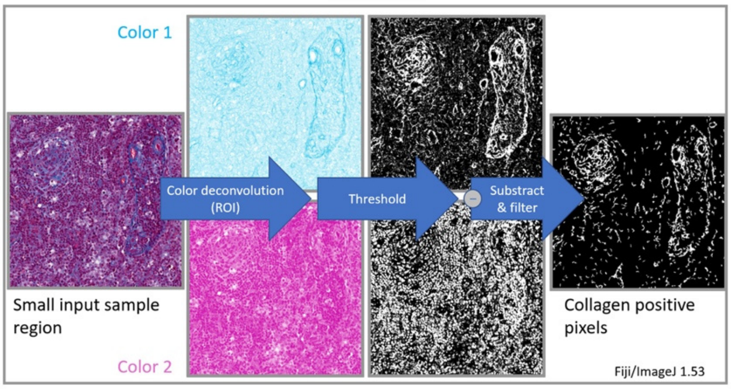

Suppl. Figure S3. Fiji pipeline for the automated quantification of collagen in tissue slices. A. Selection of a representative area within a lymph node section. B. Procedure of the Fiji pipeline applying first a colour deconvolution, conversion to a binary image using a specific threshold, substraction and filtering.

Supplementary Data

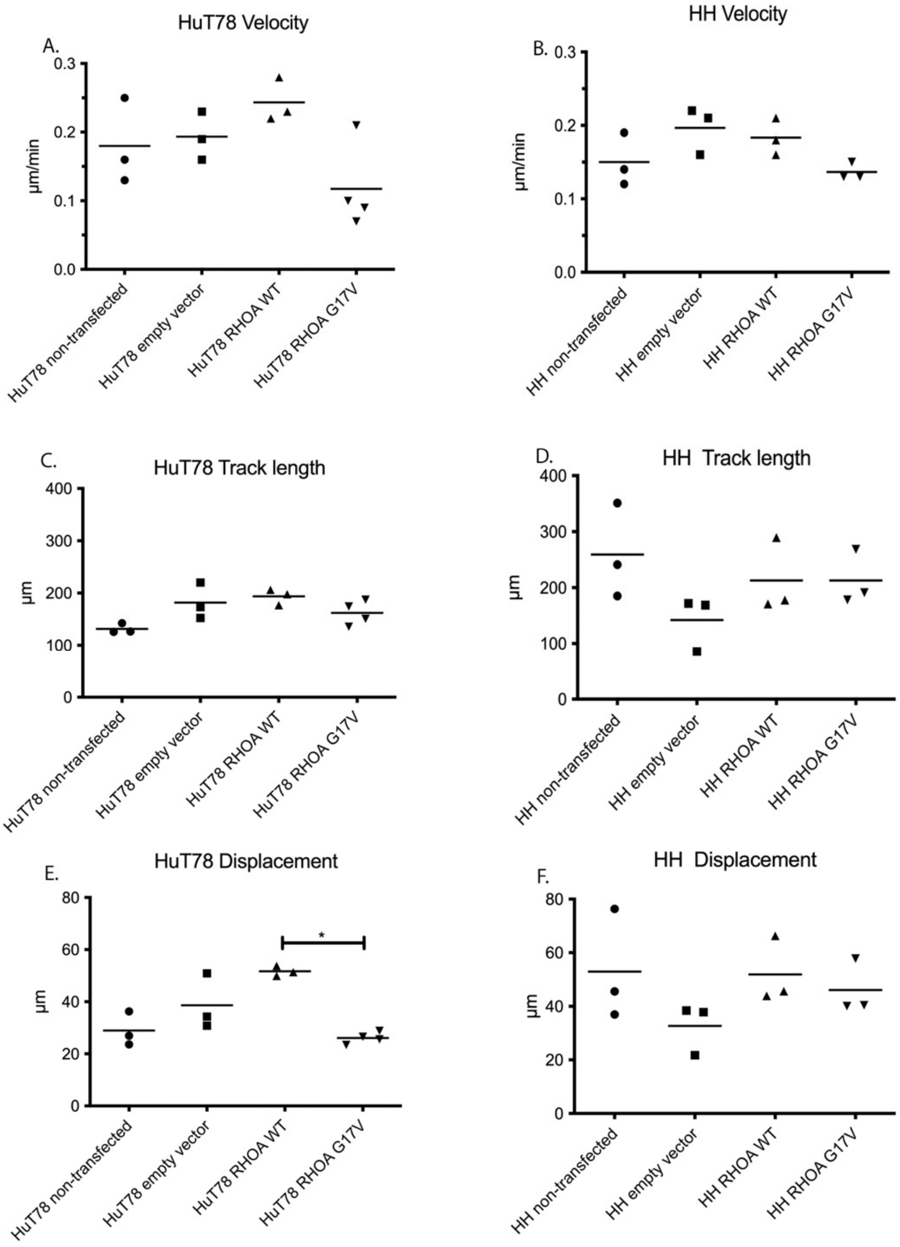

Suppl. Figure S4. Velocity, track length and displacement in a 3D collagen gel of HuT78 and HH cells untreated, empty vector transfected or transfected with vectors to stably express RHOA-WT or RHOA-G17V

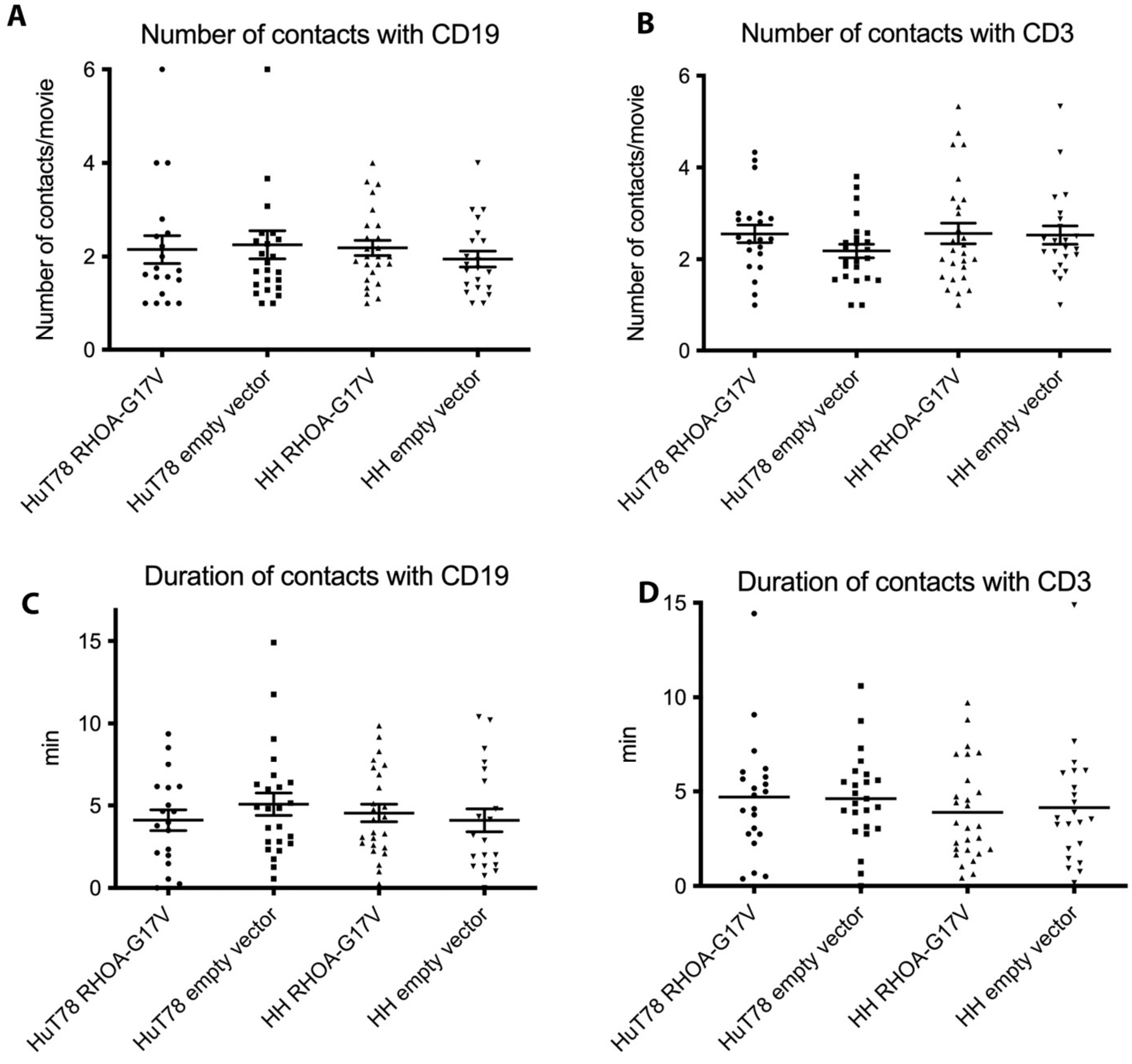

Suppl. Figure S5. Analysis of cell-cell contacts of RHOA-WT and RHOA-G17V expressing HuT78 and HH cells with endogenous tissue-resident B- (CD19) and T- (CD3) cells
A. and B. Quantification of the number of contacts (mean number of contacts/movie)
C. and D. Mean duration of contacts per movie

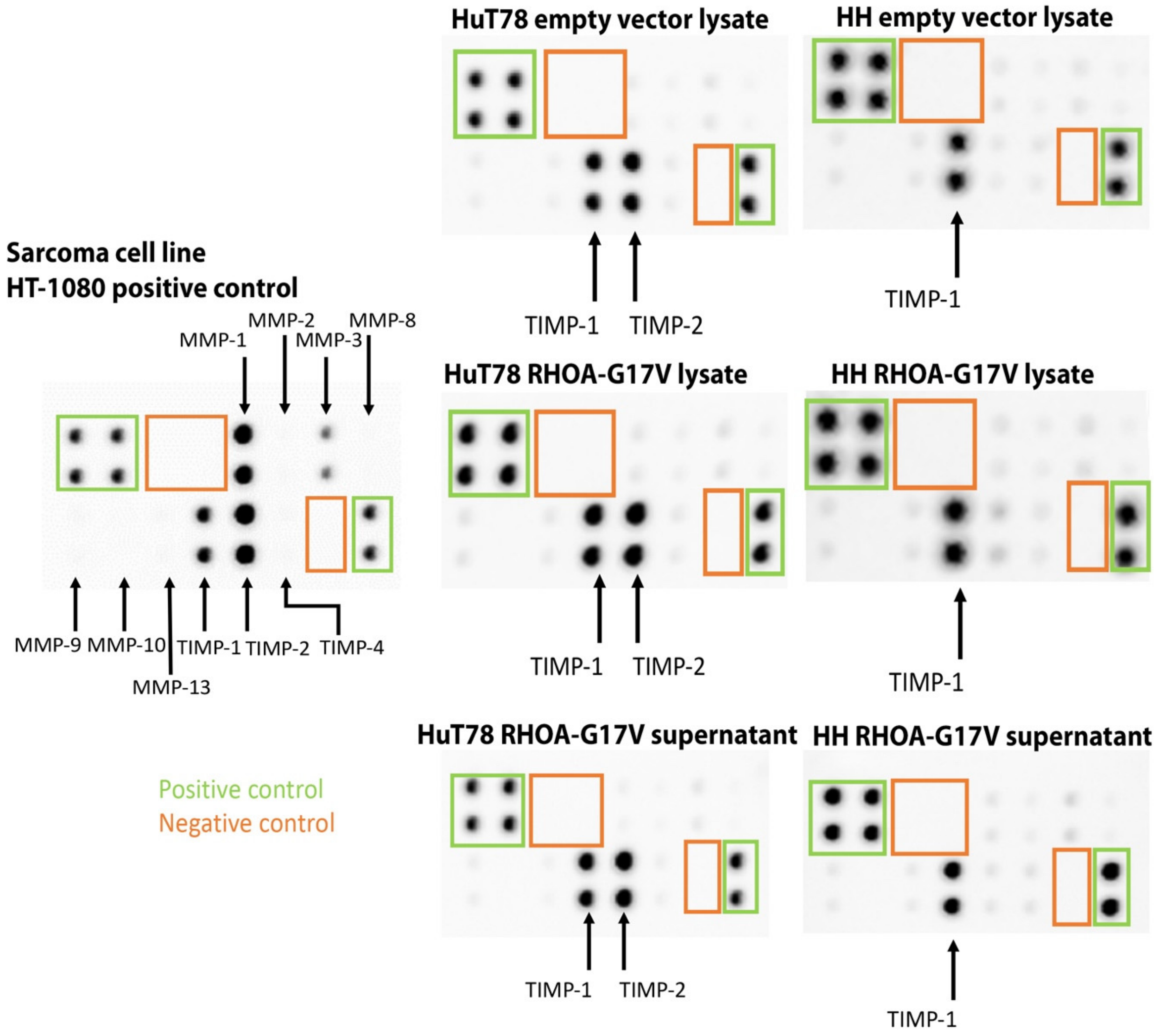

Suppl. Figure S6. MMP assay of HuT78 and HH cell lysates of empty vector transfected or RHOA-G17V expressing cells or supernatants collected from RHOA-G17V expressing cells. The sarcoma cell line HT-1080 was used as positive control.

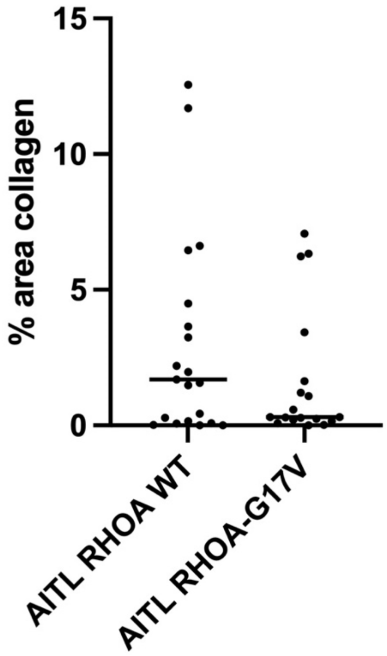

Suppl. Figure S7. Automatically quantified collagen content (area %) in AITL samples with and without RHOA-G17V mutation.

Each dot represents one case.

References

1. Kowarz, E.; Loscher, D.; Marschalek, R. Optimized Sleeping Beauty transposons rapidly generate stable transgenic cell lines. *Biotechnol J* **2015**, *10*, 647-653, doi:10.1002/biot.201400821.
2. van der Walt, S.; Schonberger, J.L.; Nunez-Iglesias, J.; Boulogne, F.; Warner, J.D.; Yager, N.; Gouillart, E.; Yu, T.; scikit-image, c. scikit-image: image processing in Python. *PeerJ* **2014**, *2*, e453, doi:10.7717/peerj.453.
